# Transplantation of mesenchymal stem cells overexpressing interleukin‐10 induces autophagy response and promotes neuroprotection in a rat model of TBI

**DOI:** 10.1111/jcmm.14396

**Published:** 2019-06-04

**Authors:** Panchanan Maiti, Sarah Peruzzaro, Nivya Kolli, Melissa Andrews, Abeer Al‐Gharaibeh, Julien Rossignol, Gary L. Dunbar

**Affiliations:** ^1^ Field Neurosciences Institute of Laboratory for Restorative Neurology Central Michigan University Mt. Pleasant Michigan; ^2^ Program in Neuroscience Central Michigan University Mt. Pleasant Michigan; ^3^ Department of Psychology Central Michigan University Mt. Pleasant Michigan; ^4^ Field Neurosciences Institute St. Mary’s of Michigan Saginaw Michigan; ^5^ Department of Biology Saginaw Valley State University Saginaw Michigan; ^6^ Brain Research Laboratory Saginaw Valley State University Saginaw Michigan; ^7^ College of Medicine Central Michigan University Mt. Pleasant Michigan

**Keywords:** autophagy markers, IL‐10 overexpressed MSCs, neuroinflammation, TBI

## Abstract

Autophagy, including mitophagy, is critical for neuroprotection in traumatic brain injury (TBI). Transplantation of mesenchymal stem cells (MSCs) provides neuroprotection and induces autophagy by increasing anti‐inflammatory cytokines, such as interleukin‐10 (IL‐10). To evaluate these effects of IL10 that are released by MSCs, we genetically engineered MSCs to overexpress IL10 and compared their effects to unaltered MSCs following transplantation near the site of induced TBIs in rats. Adult, male Sprague‐Dawley rats were divided into four groups: Sham + vehicle, TBI + vehicle, TBI + MSCs‐IL‐10 and TBI + MSCs‐GFP. Thirty‐six hours post‐TBI, the first two groups received vehicle (Hanks balance salt solution), whereas last two groups were transplanted with MSCs‐IL‐10 or MSCs‐GFP. Three weeks after transplantation, biomarkers for neurodegenerative changes, autophagy, mitophagy, cell death and survival markers were measured. We observed a significant increase in the number of dead cells in the cortex and hippocampus in TBI rats, whereas transplantation of MSCs‐IL‐10 significantly reduced their numbers in comparison to MSCs alone. MSCs‐IL‐10 rats had increased autophagy, mitophagy and cell survival markers, along with decreased markers for cell death and neuroinflammation. These results suggest that transplantation of MSCs‐IL‐10 may be an effective strategy to protect against TBI‐induced neuronal damage.

## 1 INTRODUCTION

Traumatic brain injury (TBI) is a debilitating health problem, affecting millions of people each year.^1^, ^2^ Any strong impact to the head can result in a TBI,^2^, ^3^ leading to brain damage, with subsequent motor, sensory, psychological and cognitive dysfunctions.^4^ Importantly, the damage may last for months, years or even the rest of the person's life.^5^, ^6^

Although several studies have been conducted to investigate the pathophysiology and molecular events involved in neuronal injury in TBI,^7^, ^8^, ^9^ they are still not clearly understood. Inflammation is one of the main causes of neuronal damage in TBI.^10^, ^11^ Interestingly, during TBI, M1‐subtype of microglia becomes activated, which releases significant amount of pro‐inflammatory cytokines, such as tumour necrosis factor‐α (TNF‐α), interferon gamma (IFN‐γ), IL‐1β, IL‐6, IL‐12 12 and reactive oxygen species (ROS), that disrupt the blood brain barrier (BBB) 13 and trigger neuronal injury.^14^ In contrast, M2‐subtype of microglia releases anti‐inflammatory cytokines, such as IL‐10, IL‐4, IL‐13, tumour growth factor (TGF) that promotes wound healing and the reduction in neuroinflammation.^15^ In addition to being released by M2‐microglia, IL‐10 is also secreted by mesenchymal stem cells (MSCs), as well as by neighboring neurons.^17^ IL‐10 inhibits the production and release of pro‐inflammatory cytokines,^17^ inhibits astrocyte activation and increases the expression of excitatory amino acid transporter‐2 (EAAT2), thus reducing the glutamate excitotoxicity,^18^ as noted in animal models of spinal cord injury,^19^ stroke and TBI.^17^

Several research reports suggested that MSC transplantation plays beneficial roles in the treatment of TBI,^20^ by releasing anti‐inflammatory chemokines, which can reduce neuronal injury.^21^ To augment the MSCs release of anti‐inflammatory cytokines,^22^ the use of genetically engineered MSCs that overexpress IL‐10 (MSCs‐IL‐10), delivered to injured brain areas.^23^, ^24^ Recently, we found that the transplantation of MSCs‐IL‐10 promotes conversion of M1 to M2 macrophages, thereby increasing anti‐inflammatory cytokines and decreasing inflammatory responses in rats given TBI.^24^ In this study, we used the Morris water maze (MWM), ladder‐rung walking and rotarod tasks to assess cognitive function, inter‐limb coordination, and locomotor abilities, respectively. Although all rats acquired the MWM task equally, the mean latency to find the hidden platform was reduced in TBI + MSCs‐IL‐10 transplanted rats during the reversal sessions, suggesting an IL‐10‐induced reduction in TBI‐induced deficits in learning and memory. Similarly, we also found that both MSCs‐IL‐10 and MSCs rats had reduced motor deficits on the ladder‐rung walking test, but not on the rotarod test in rats.^24^

One of the mechanisms for MSCs‐transplantation‐induced neuroprotection could involve the induction of autophagy mechanisms.^25^ Autophagy is the cellular active clearance mechanism, which interacts with apoptotic pathways and helps to determine the cell fate.^26^, ^27^, ^28^ Importantly, transplantation of MSCs significantly enhances autophagy in animal models of AD, which increases neuronal survival against toxic amyloid proteins.^29^, ^30^, ^31^

In the present study, we investigated the levels of autophagy, mitophagy, molecular chaperones, neuroinflammation, cell death and synaptic functioning in rats given TBI and treated with either vehicle, transplanted MSCs or transplanted MSCs which were genetically modified to overexpress IL‐10 (MSCs‐IL‐10). We observed that MSCs‐IL‐10 rats improved neuronal morphology, reduced neurodegeneration and diminished number of DNA‐fragmented cells, compared with rats transplanted with unaltered MSCs. Furthermore, MSCs‐IL‐10 rats had increased autophagy, markers for mitophagy, cell survival and pre‐ and post‐synaptic function while reduced levels of cell death markers than rats given unaltered MSCs.

## 2 MATERIAL AND METHODS

### 2.1 Chemicals

Cresyl violet, polybrene, puromycin, Hank's balanced salt solution (HBSS) and other accessory chemicals were procured from Sigma (St. Louis, MO). Fluoro‐Jade B (FJB) stain was purchased from Millipore (Burlington, MA. Terminal deoxyribonucleic acid nick end labeling (TUNEL) kit was from Abcam (Cambridge, MA). Polyvinylidene difluoride (PVDF) membrane was from Molecular Probe (Grand Island, NY). Hoechst 33342 solution (20 mmol L^−1^) and 293FT cell lines were purchased from ThermoFisher Scientific (Grand Island, NY). pGEM‐T Easy Vector was from Promega (Fitchburg, WI). The control plasmid, pLenti‐CMV‐GFP‐2A‐Puro and pLenti‐CMV‐GFP‐2A‐Puro vectors were purchased from Applied Biological Materials Inc (Richmond, BC, Canada). The information for different antibodies used for this study is provided in Table 1.

**Table 1:** Sources of different antibodies used in this study

Antibodies	Source	Type	Company	Catalogue no.	Address
IL‐10	Rabbit	Polyclonal	Cell signaling Technology	12163	Danvers, MA
Iba‐1	Rabbit	Polyclonal	Wako	019‐19741	Richmond, VA
GFAP	Rabbit	Monoclonal	Cell signaling Technology	12389	Danvers, MA
HSP90	Rabbit	Monoclonal	Cell signaling Technology	4877	Danvers, MA
HSP70	Rabbit	Monoclonal	Cell signaling Technology	4872	Danvers, MA
HSP60	Rabbit	Monoclonal	Cell signaling Technology	12165	Danvers, MA
HSC70	Rabbit	Monoclonal	Cell signaling Technology	8444	Danvers, MA
HSP40	Rabbit	Monoclonal	Cell signaling Technology	4871	Danvers, MA
CHIP	Rabbit	Monoclonal	Cell signaling Technology	2080	Danvers, MA
Atg5	Rabbit	Monoclonal	Cell signaling Technology	12994	Danvers, MA
Atg7	Rabbit	Monoclonal	Cell signaling Technology	8558	Danvers, MA
Beclin‐1	Rabbit	Polyclonal	Cell signaling Technology	3738	Danvers, MA
LC3A/B	Rabbit	Monoclonal	Cell signaling Technology	12741	Danvers, MA
p62	Rabbit	Monoclonal	Cell signaling Technology	5114S	Danvers, MA
mTOR	Rabbit	Monoclonal	Cell signaling Technology	2983	Danvers, MA
p‐mTOR	Rabbit	Polyclonal	Cell signaling Technology	2971	Danvers, MA
LAMP2A	rat	Monoclonal	Santa Cruz Biotechnology	sc‐8100	Santa Cruz, CA
NIX	Rabbit	Monoclonal	Cell signaling Technology	12396	Danvers, MA
BNIP3	Mouse	Monoclonal	Santa Cruz Biotechnology	Sc‐56167	Santa Cruz, CA
PINK1	Rabbit	Monoclonal	Cell signaling Technology	6946S	Danvers, MA
FUNDC1	Rabbit	Polyclonal	Abcam	ab74834	Cambridge, MA
HIF‐1α	Rabbit	Monoclonal	Cell signaling Technology	14179	Danvers, MA
pAkt (Ser473)	Rabbit	Monoclonal	Cell signaling Technology	9271	Danvers, MA
Akt	Rabbit	Polyclonal	Cell signaling Technology	9272	Danvers, MA
PI3K	Rabbit	Polyclonal	Cell signaling Technology	4292S	Danvers, MA
PSD95	Rabbit	Polyclonal	Santa Cruz Biotechnology	sc‐71933	Santa Cruz, CA
Synaptophysin	Rabbit	Mouse	Cell signaling Technology	12270S	Danvers, MA
p53	Rabbit	Polyclonal	Cell signaling Technology	9282	Danvers, MA
Bcl2	Mouse	Monoclonal	Santa Cruz Biotechnology		Santa Cruz, CA
Bax	Rabbit	Polyclonal	Cell signaling Technology	2772S	Danvers, MA
Cytochrome‐C	Rabbit	Monoclonal	Cell signaling Technology	11940S	Danvers, MA
Caspase‐3	Rabbit	Polyclonal	Cell signaling Technology	9662S	Danvers, MA
β‐tubulin	Rabbit	Monoclonal	Cell signaling Technology	15115	Danvers, MA

### 2.2 Animals

Thirty‐nine male, Sprague‐Dawley (SD) rats (Charles River, Mattawan, MI) approximately 90 days old, were used in this study. Rats were paired housed in a 12h/12h reverse light cycle with food and water ad libitum. Rats were randomly divided into four groups: Sham + HBSS (n = 10), TBI + HBSS (n = 10), TBI + MSCs‐IL‐10 (CMV‐IL‐10‐GFP, n = 9) and TBI + MSCs (CMV‐GFP, n = 10). All procedures were approved by the Institutional Animal Care and Use Committee at Central Michigan University.

### 2.3 Rat model of tbi using controlled cortical impactor

The method for inducing the TBI was described previously.^24^, ^32^ Briefly, the rats were anaesthetized using a mixture of 1%‐3% isoflurane (Henry Schein Co.) and 500 mL‐L/min oxygen and maintained throughout the surgery. Body temperature was maintained at 37°C during surgeries using a physitemp machine (Physitemp Instruments Inc Clifton, NJ). Rats were placed on a stereotaxic instrument (Kopf Instruments, Tujunga, CA) and a midline incision was made to expose bregma. Sham rats were given an incision which exposed the skull, without causing the TBI. Rats in the injured groups then underwent a 6‐mm craniotomy at 3 mm anterior to bregma (AP + 3.0, ML 0.0 mm). The impactor tip was placed over the exposed brain and compressed the cortex at a depth − 2.5 mm at a velocity of 2.25 m/s with a duration of 0.5 seconds.^32^ The upper skin of the head was stitched and allowed to recover.

### 2.4 Lentivirus construction for il‐10

The detailed method for lentivirus construction for IL‐10 was described by Peruzzaro and colleagues.^24^

### 2.5 Isolation of mesenchymal stem cells

The MSCs were isolated and cultured as described previously.^24^, ^33^ Viral production and their expression were confirmed from puromycin (10 µg/mL)‐selected colonies. Flow cytometry and immunocytochemistry (ICC) were performed to confirm MSCs surface markers and viral transfection.^24^

### 2.6 Stem cell transplantation

Transplantation surgery was performed 36 hours after injury, as described previously.^36^ Sham + HBSS and TBI + HBSS rats were injected with HBSS, whereas TBI + MSCs‐IL‐10 rats were injected with MSCs‐IL‐10 and TBI + MSCs‐GFP rats were transplanted with MSCs‐GFP.

### 2.7 Tissue processing

Three weeks after transplantation, all rats were deeply anaesthetized with an overdose of sodium pentobarbital (intraperitoneally) and transcardially perfused with 0.1 mol L^−1^ cold PBS, followed by 4% paraformaldehyde (diluted in 0.1 mol L^−1^ PBS at pH 7.4) to fix the brains. The brains were then removed, suspended in 4% paraformaldehyde for 24 hour at 4°C and then transferred to the graded sucrose solutions (10%, 20% and 30%), dissolved in 0.1 mol L^−1^ PBS, and then frozen using 2‐methylbutane and stored in the −80°C freezer until they were sectioned coronally (30 µm) on a cryostat (Vibratome UltraPro 5000) set at −20°C. Brains from rats used for Western blot analysis were directly removed without perfusion and the fresh tissue was flash‐frozen using 2‐methylbutane (Sigma) and stored at −80°C until further use.

### 2.8 Neuronal morphology by cresyl violet staining

The rat brains from all four groups were sectioned coronally on a cryostat (Leica, Germany) and then they were stained with 0.1% Cresyl violet (CV) as described previously.^41^ The sections were then washed, dehydrated, cleared and mounted by cover slip using DePex mounting media (BDH, Batavia, IL). The slides were dried and the photomicrographs were taken by compound light microscope (Olympus, Japan) using 100×objectives (total magnification of 1000×). Dark, large dot stained cells were considered as pyknotic or tangle‐like cells were counted manually using Image‐J software (http://imagej.nih.gov/ij) and expressed as number of pyknotic cells per 1 mm^2^ area. A minimum of 10 serial sections, with 30 different fields was used to count the number of pyknotic cells in each group (n = 6). Two researchers counted the cells separately and an average value was reported.

### 2.9 Neurodegeneration study by Fluoro‐Jade B (FJB) staining

Ten coronal sections (at equal interval from bregma + 2.20 mm to 0.70 mm for cortex and −2.20 mm to −3.60 mm for hippocampus) were cut on a cryostat (20 μm) and placed in 0.1 mol L^−1^ PBS, mounted on gelatin‐coated (2%) slides and then air dried on a slide warmer at 50°C for 30 minutes. The protocol used for FJB staining was described previously.^34^ The total number of FJB‐positive cells were counted using Image‐J software (http://imagej.nih.gov/ij), expressed per 1 mm^2^ area. A minimum of 10 serial sections, with 20 different fields was used to count the number of FJB‐positive cells in each group (n = 6). All experimenters were blinded to the group identity of the specimens analysed.

### 2.10 Terminal deoxyribonucleic acid nick end labeling (tunel)

Coronal brain sections (20 µm) from each of the group of rats were taken in polylysine‐coated glass slide and TUNEL staining was performed as described previously.^35^, ^36^ All sections were counterstained with Hoechst‐33342 (20 mmol L^−1^) for 5 minutes at room temperature in the dark and washed thoroughly with distilled water before being mounted on a glass slide with anti‐fading medium (Sigma). The cells were counted using a fluorescent microscope (Leica, Germany) with appropriate filters (ex/em: 488/576) so that TUNEL‐positive cells flourished red. The number of TUNEL‐positive cells was counted manually using ImageJ software as reference frame (http://imagej.nih.gov/ij) from three experiments to obtain a mean value of cells per 1 mm^2^ area.

### 2.11 Immunohistochemistry of Atg5, Atg7

Immunoperoxidase techniques were used for the levels of Atg5 and Atg7. Briefly, cryosections (40‐μm thick) were rinsed with PBS (0.1 mol L^−1^, at pH 7.4) three times and then incubated with 0.5% Triton‐X100, along with 3% H_2_O_2_ solution for 30 minutes at room temperature, followed by three washes in PBS, for 10 minutes each. The unmasking was done by treating the sections with 10% normal goat serum for 1 hour at room temperature. Then the sections were incubated with rabbit monoclonal anti‐Atg5 anti‐Atg7 antibodies (1:200), which were dissolved in PBS, along with 10% goat serum and placed on the plate on a shaker at low speed and kept at 4°C overnight. On the next day, the sections were thoroughly washed with PBS, three times for 10 minutes each. The sections were incubated with biotinylated anti‐rabbit secondary antibody (Vector Laboratory, CA; 1:200) for 4 hour at 37°C. After this incubation, the sections were washed three times with PBS, 10 minutes each and then treated with ABC reagent for 30 minutes at room temperature. This was followed by three washes in PBS for 10 minutes each. Finally, the sections were incubated with peroxidase substrate solution, supplied with the ABC kit (Vector Laboratory, CA) and the signal was developed using diaminobenzidine (DAB) until the desired staining intensity emerged. The tissue was then washed, dehydrated, cleared, mounted on slides and visualized using a compound light microscope (Olympus, Japan).

### 2.12 Confocal imaging of GFAP, Iba‐1, Beclin‐1 and LC‐3A/B

Immunofluorescent technique was used for detecting levels of Beclin‐1, LC‐3A/B, GFAP and Iba‐1, as described previously.^39^ Briefly, after blocking with normal goat serum (10%), the sections were incubated overnight with Beclin‐1 (1:200), LC‐3A/B (1:200), GFAP (1:1000) and Iba‐1 (1:4000) antibodies (1:200, Table 1). On the following day, the tissue was incubated with anti‐rabbit secondary antibody (1:500), tagged with FITC (for Beclin‐1, Molecular Probes, OR) or Alexa‐594 (for LC‐3A/B) for 30 minutes at room temperature and then washed thoroughly with distilled water, dehydrated, cleared and mounted on slides using anti‐fading fluoro‐mount aqueous mounting media (Sigma). Using a tabletop Fluoview confocal laser scanning microscope (FV1oi, Olympus) with appropriate filters to optimize excitation and emission, the number of GFAP‐IR and Iba‐1‐IR cells was counted around the lesion site, manually, using Image‐J software from 10 sections in the cortex, CA1 and CA3 subfield of hippocampus and expressed as number of GFAP‐IR or Iba‐1‐IR/1 mm^2^ area.

### 2.13 Western blots

About 100 mg of flash‐frozen mixed cortex was lysed with cold radioimmunoprecipitation assay (RIPA) buffer with protease and phosphatase inhibitors (Sigma), as described previously.^39^ After probing with respective primary and secondary antibodies (Table 1), the blots were developed with ImmobilonTM Western Chemiluminescent HRP substrate (Millipore, Billeria, MA). The relative optical density was measured using Image‐J software (https://imagej.nih.gov/ij/). To ensure equal protein loading in each lane, the blots were re‐probed for β‐tubulin.

### 2.14 Statistical analysis

All data are expressed as Mean ± SEM. All statistics were analysed using ANOVA, followed by post‐hoc Tukey's Honestly Significant Difference (HSD) test. The probability value *P* ≤ 0.05 was considered significant.

## 3 RESULTS

Using IHC, RT‐PCR and Western blot techniques, we previously confirmed the overexpression of IL‐10 levels in MSCs‐IL‐10 cells in vitro and in vivo.^24^ In addition, we have also confirmed an increase in levels of IL‐10 in TBI rats transplanted with MSCs‐IL‐10 over the other three groups 24 (Figure S1).

### 3.1 Transplantation of mscs‐il‐10 protected cortical and hippocampal neuronal damage better than transplantation of mscs alone in tbi rats

**Figure 1:**
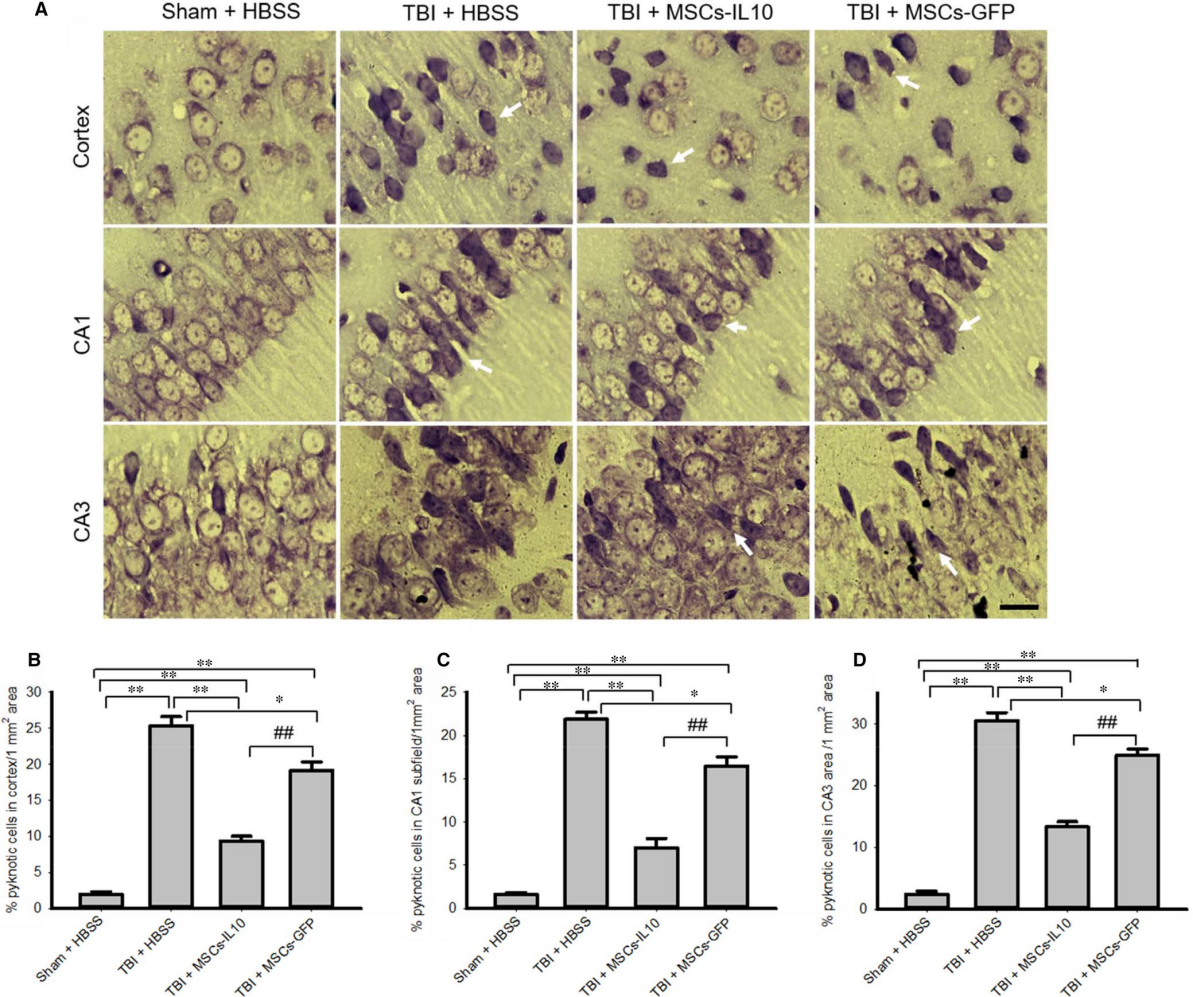
Transplantation of MSCs‐IL‐10 improved neuronal morphology greater than MSCs alone in the cortex and hippocampus of TBI rats. Rat brains were sectioned and stained with 0.1% Cresyl violet and images were taken by compound light microscope (Olympus) with 100× objectives (total mag 1000×). (A) Representative photomicrograph of TBI rats showed increase in number of pyknotic or tangle‐like cells in the cortex, in the CA1 and CA3 subfields of hippocampus. (B‐D) Number of pyknotic cells were significantly decreased by transplantation of MSCs‐IL‐10 in comparison to TBI rats (*P* < 0.01) and with TBI + MSCs (*P* < 0.01). The greater reduction in pyknotic cells was observed in the case of MSCs‐IL‐10 rats. Arrows indicate pyknotic or tangle‐like cells. Scale bar indicates 100 µm and is applicable to other images. **P < 0.01 in comparison to TBI + HBSS, TBI + MSCs‐IL‐10 and TBI + MSCs; *P < 0.05 in comparison to TBI + MSCs; ^##^ *P* < 0.01 in comparison to TBI + MSCs

To characterize the morphological changes after TBI morphology, we stained coronal sections with 0.1% Cresyl violet. We observed that the number of pyknotic or tangle‐like cells was significantly increased (*P* < 0.01) in the cortex (B), in CA1 (C) (*P* < 0.01) and CA3 (D) (*P* < 0.01) subfields of hippocampus of TBI rats (Figure 1A‐D) and that these pyknotic cells were significantly decreased (*P* < 0.01) in rats which received MSCs‐IL‐10 cells. Greater reduction in pyknotic or tangle‐like cells was observed in these areas in the case of MSCs‐IL‐10‐treated rats in comparison of MSCs alone (*P* < 0.01).

### 3.2 Transplantation of mscs‐il‐10 protected against tbi‐induced than mscs alone

**Figure 2:**
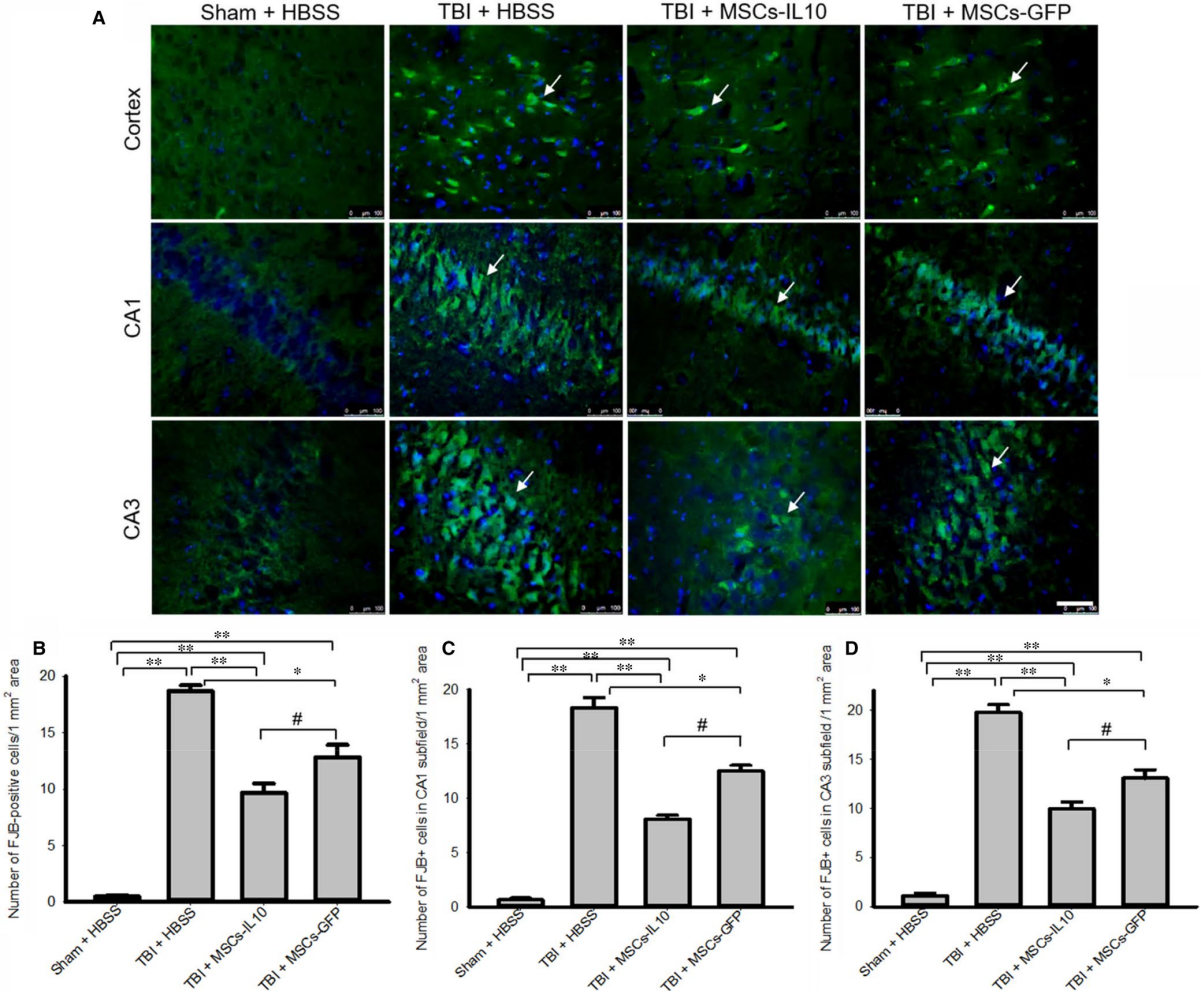
Transplantation of MSCs‐IL‐10 reduced the number of degenerated neurons in the cortex and hippocampus of TBI rats than MSCs alone. Rat brains were sectioned (20 µm) and stained with Fluoro‐Jade B (FJB) solution (0.0004%) and images were taken using fluorescent microscope (Leica, Germany). (A) Representative images of FJB‐stained sections from the cortex, in the CA1 and CA3 area of hippocampus. (B‐D) The number of FJB‐positive cells was significantly increased (**P < 0.01) in TBI rats, whereas transplantation of both MSCs‐IL‐10 and MSCs alone, significantly decreased the number of FJB‐positive cells. The number of FJB cells was significantly less (^#^ *P* < 0.05) in the case of TBI + MSCs‐IL‐10 in comparison to TBI + MSCs. Green signals (red arrows) indicate FJB‐positive cells and blue signal is for DAPI (nuclear) stain. Scale bar indicates 100 µm and is applicable to other images. **P < 0.01 in comparison to TBI + HBSS, TBI + MSCs‐IL‐10 and TBI + MSCs; *P < 0.05 in comparison to TBI + MSCs; ^#^ *P* < 0.05 in comparison to TBI + MSCs

To characterize the neuronal injury in TBI model, the coronal sections were stained with FJB (Figure 2A), a marker for neurodegeneration. The number of FJB‐positive cells was significantly increased in the cortex (B), in the CA1 (C) and CA3 (D) subfields of hippocampus in TBI group (*P* < 0.01), whereas transplantation of both MSCs‐IL‐10 and MSCs‐GFP significantly (*P* < 0.05) decreased the number of FJB‐positive cells (B‐D), however, MSCs‐IL‐10 decreased more degenerated cells than MSCs‐GFP alone (*P* < 0.05).

### 3.3 Number of dna‐fragmented cells were reduced more by transplantation of mscs‐il‐10 than mscs alone

**Figure 3:**
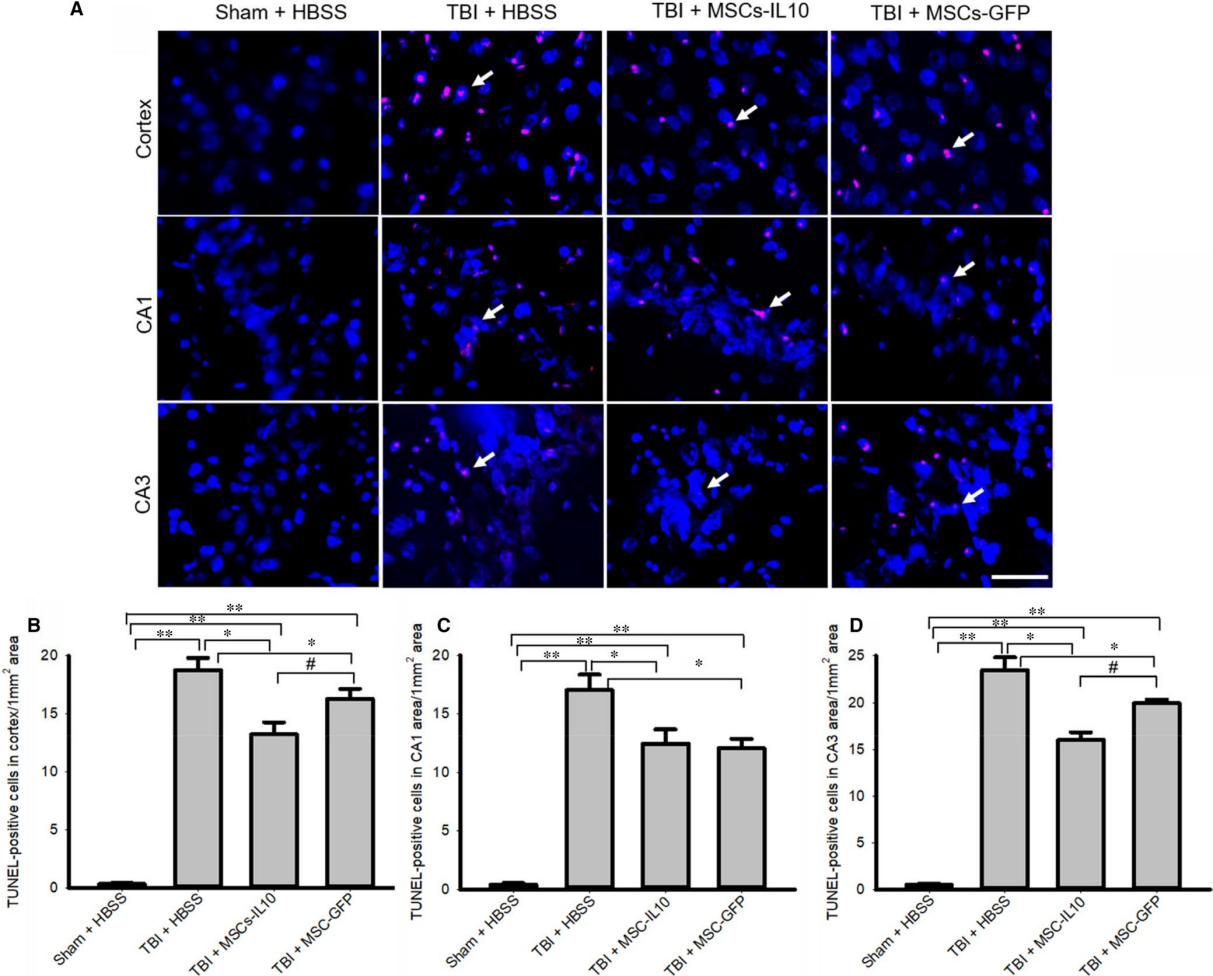
Transplantation of MSCs‐IL‐10 reduced greater number of DNA‐fragmented cells in the cortex and in the hippocampus of TBI rats than MSCs alone. Terminal deoxyribonucleic acid nick end labeling (TUNEL) was performed in coronal sections from cortex, CA1 and CA3 subfields of hippocampus. (A) Representative photomicrographs showed an increase in number of TUNEL‐positive cells in the cortex, as well as in the CA1 and CA3 areas of the hippocampus. (B‐D) The number of TUNEL‐positive cells was significantly increased (**P < 0.01) in TBI rats, whereas transplantation of both MSCs‐IL‐10 and MSCs alone, significantly decreased their numbers. The number of TUNEL‐positive cells was significantly less (^#^ *P* < 0.05) in the case of TBI + MSCs‐IL10 in the cortex and CA3 area of hippocampus in comparison to TBI + MSCs. Red signals (white arrows) indicate TUNEL‐positive cells and blue signal is for DAPI (nuclear) stain. Scale bar indicates 100 µm and is applicable to other images. **P < 0.01 in comparison to TBI + HBSS, TBI + MSCs‐IL‐10 and TBI + MSCs; *P < 0.05 in comparison to TBI + HBSS; ^#^ *P* < 0.05 in comparison to TBI + MSCs

To examine the mode of cell death in TBI after transplantation with MSCs‐IL‐10 or MSCs‐GFP, we performed TUNEL staining of the tissue from the cortex, CA1 and CA3 areas of hippocampus (Figure 3). We observed that TBI group significantly increased the number of TUNEL‐positive cells in the cortex (Figure 3A‐B), in the CA1 (Figure 3A and C) and CA3 areas (Figure 3A and D) of hippocampus in comparison to Sham + HBSS (*P* < 0.01). Whereas transplantation of MSCs‐IL‐10 and MSCs alone significantly decreased (*P* < 0.05) their levels. However, MSCs‐IL‐10 rats had fewer TUNEL‐positive cells than rats receiving MSCs alone (*P* < 0.05).

### 3.4 Transplantation of mscs‐il‐10 modulated autophagy markers and pi3k/akt/mtor pathway greater than unaltered mscs in tbi rats

**Figure 4:**
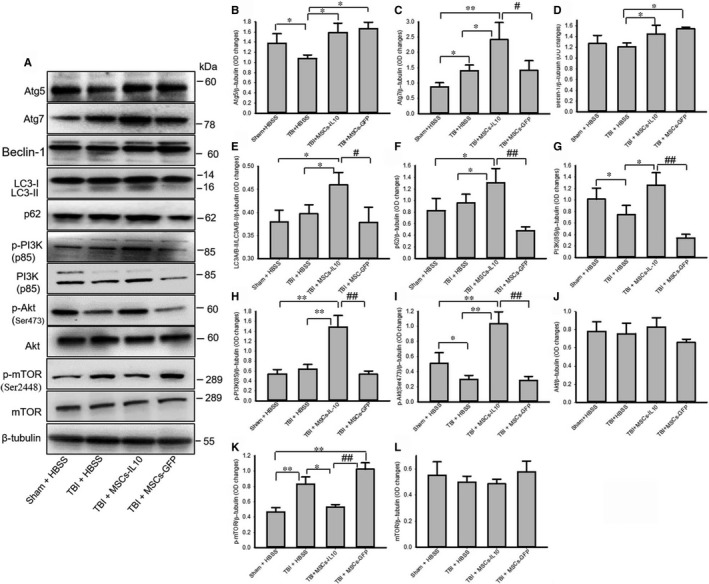
Transplantation of MSCs‐IL‐10‐modulated autophagy markers and PI3K/Akt/mTOR pathway greater than MSCs alone in the TBI rats. Equal amount of protein from cortical tissue homogenates was electrophoresed, blotted on PVDF membrane and different autophagy markers were studied. (A) Representative Western blots of Atg5, Atg7, Beclin‐1, LC3A/B, p62, PI3K, p‐Akt, Akt, p‐mTOR, mTOR from mixed cortical tissue from different animal groups. (B‐C) Densitometric data indicating that TBI with MSCs‐IL‐10 and MSCs groups of rats showed an increase in Atg5 (B) and Atg7 (C) in comparison to TBI + HBSS or Sham + HBSS rats and greater increase was noted in the case of MSCs‐IL‐10 rats in comparison to MSCs alone. (D‐E) Western blot data showed that there was a decrease in Beclin‐1 levels in the TBI rats, which was restored by transplantation of MSCs‐IL‐10, but not by MSCs alone. (E‐F) Western blot data showed that there was an increase in levels of in LC‐3A/B‐II and p62 in TBI rats transplanted with MSCs‐IL‐10, but not by MSCs alone. (G‐I) PI3K (p85) and p‐PI3K (p85) and p‐Akt levels were less in TBI rats and they were restored by MSCs‐IL‐10, not by MSCs alone. (K) p‐mTOR levels were increased by TBI rats and restored by MSCs‐IL‐10, not by MSCs alone. *P < 0.05 and **P < 0.01 in comparison to other groups; ^#^ *P* < 0.05, ^##^ *P* < 0.01 in comparison to MSCs alone

We have observed that autophagy markers Atg5 (Figure 4A‐B), Atg7 (Figure 4A and C), LC3A/B‐II (Figure 44A, D) and p62 (Figure 4A and F) were increased greater in TBI rats which received MSCs‐IL‐10, than by MSCs alone. Whereas, there was a significant decrease in levels of PI3K (p85) and p‐Akt which were restored by transplantation of MSCs‐IL‐10 and not by MSCs (Figure 4 A, G, H and I). Although p‐PI3K (p85) was unchanged in TBI rats, it was increased only by MSCs‐IL‐10 (Figure 4A and H). In contrast, p‐mTOR (ser2448) was significantly increased by TBI and it was restored only after transplantation of MSCs‐IL‐10, (Figure 4A and K), whereas there was no meaningful change in total Akt and mTOR levels (Figure 4A and L) any group.

### 3.5 Transplantation of mscs‐il‐10 showed greater immunoreactivity of autophagy markers in tbi rats than unaltered mscs

**Figure 5:**
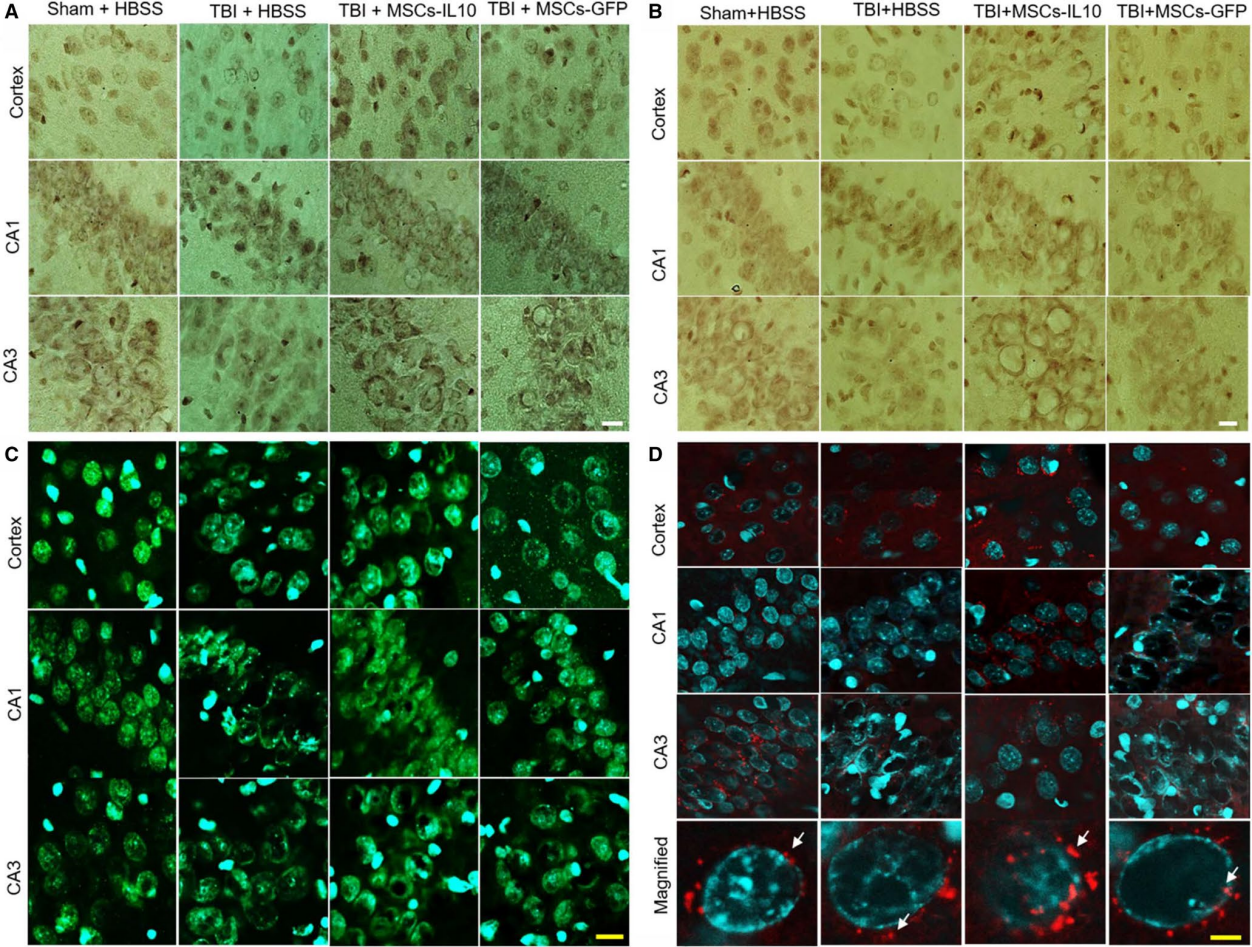
Immunohistochemistry of autophagy markers in TBI rats after transplantation of MSCs‐IL‐10 and MSCs‐GFP cells. Coronal sections from each group were immunolabelled with Atg5, Atg7, Beclin‐1 and LC3A/B antibodies. The images were taken by either light microscope (Olympus) or by tabletop Fluoview confocal laser scanning microscope (FV1oi, Olympus). Atg5 (A) and Atg7 (B) appeared to increase their levels in TBI rats after transplantation of MSCs‐IL‐10 and MSCs‐GFP when compared to TBI and sham controls. Whereas, TBI section appeared to contain less Beclin‐1 immunofluorescent signal in the TBI rats when compared to sham control or the other transplanted groups (C). Furthermore, TBI rats showed relatively less immunofluorescent puncta of LC3A/B (D) in comparison to TBI rats, whereas its level was increased after transplantation of MSCs‐IL‐10 cells and by transplants of MSCs‐GFP cells in comparison to sham control and TBI rats. Arrows indicate LC‐3A/B immunoreactivity. Blue colour: Hoechst‐3442 and green colour: secondary antibody tagged with Alexa fluoro‐488. Scale bar indicates 50 µm and applicable to other images

Following TBI, the immunointensity of Atg5 (Fig 5A) and Atg7 (Fig 5B) appeared to increase in the cortex and hippocampus of the MSCs‐IL10 and MSCs‐GFP rats, but not the TBI + HBSS and Sham + HBSS rats. However, we did not observe any meaningful changes between Sham + HBSS and TBI + HBSS rats in these areas. We observed decreased levels of immunofluorescent signal of Beclin‐1 in TBI rats, but only transplantation of MSCs‐IL‐10, appeared to further increase these levels (Figure 5C). In addition, immunofluorescent signal for LC3A/B was intensified in MSCs‐IL‐10 rats in comparison to all other groups (Figure 5D).

### 3.6 Mitophagy markers were increased more in mscs‐il‐10 rats than in those with mscs alone

**Figure 6:**
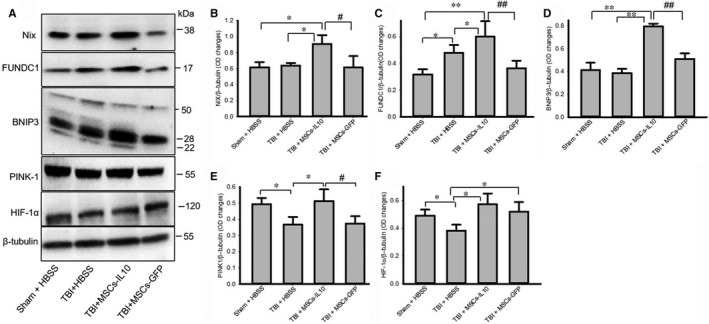
Transplantation of MSCs‐IL‐10, but not MSCs alone increased mitophagy markers in TBI rats. Western blot analyses showed that mitophagy markers, such as NIX, FUNDC1 and BNIP3 were unaltered, whereas PINK‐1 and HIF‐1α were down‐regulated by TBI rats and transplantation of MSCs‐IL‐10, but not transplantation of MSCs alone improved their levels. *P < 0.05 and **P < 0.01 in comparison to TBI + HBSS and Sham + HBSS and TBI + MSCs‐GFP rats; ^#^ *P* < 0.05 and ^##^ *P* < 0.01 in comparison to TBI + MSCs

Although alterations of NIX, FUNDC1 and BNIP3 in TBI rats were minimal, transplantation of MSCs‐IL‐10, but not MSCs alone significantly increased (*P* < 0.05) their levels (Figure 6A‐D). In contrast, PINK‐1 and HIF‐1α levels were significantly down‐regulated by TBI rats (*P* < 0.05) and levels of both these proteins were restored by MSCs‐IL‐10, whereas transplantation of MSCs restored HIF‐1α levels (Figure 6A and F), but not the PINK‐1 levels (Figure 6A and E).

### 3.7 Transplantation of mscs‐il‐10, but not mscs alone, increased cell survival markers and reduced cell death markers in tbi rats

**Figure 7:**
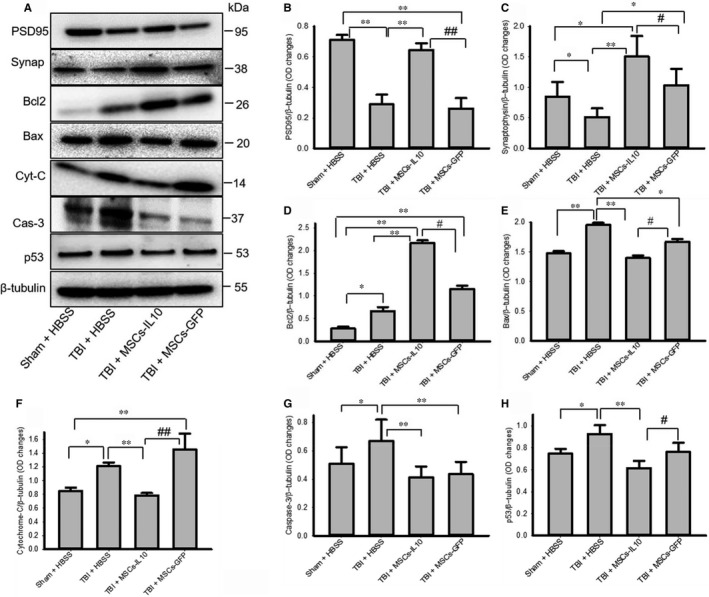
Transplantation of MSCs‐IL‐10, but not MSCs‐GFP improved synaptic and cell survival markers and decreased cell death markers in TBI rats. (A‐C) Pre‐synaptic and post‐synaptic markers, such as synaptophysin and PSD95 were down‐regulated by TBI and transplantation of MSCs‐IL‐10 and MSCs alone improved both, but MSCs‐IL‐10 improved greater than MSCs alone. (D) Anti‐apoptotic marker Bcl2 was increased greater by MSCs‐IL10, than MSCs alone. (E‐H) Whereas cell death markers, such as Bax, cytochrome‐C, caspase‐3 and p53 levels were increased by TBI and transplantation of MSCs‐IL10, decreased their levels more effectively than by MSCs alone. *P < 0.05 and **P < 0.01 in comparison to Sham + HBSS rats, ^#^ *P* < 0.05 and ^##^ *P* < 0.01 in comparison to TBI + MSCs

The levels of PSD95 (Figure 7A‐B) and synaptophysin (Figure 7A and C) were significantly decreased (*P* < 0.01) in TBI rats and transplantation of MSCs‐IL‐10, but not MSCs alone restored both the PSD95 and synaptophysin levels. In addition, cell death markers, such as Bax, cytochrome‐C, caspase‐3 and p53 levels were significantly increased (*P* < 0.05) in TBI rats, but transplantation of MSCs‐IL‐10 decreased their levels, while transplantation of MSCs alone only down‐regulated caspase‐3 and p53 levels (Figure 7A and E‐F). In contrast, Bcl2 levels were significantly increased by MSCs‐IL‐10 and MSCs alone in comparison to Sham + HBSS and TBI + HBSS rats, with Bcl2 levels being significantly higher (*P* < 0.05) in the case of MSCs‐IL‐10 in comparison to MSCs alone (Figure 7A and D).

## 4 DISCUSSION

Traumatic brain injury (TBI) is one of the leading causes of motor, sensory, psychological and cognitive dysfunction. It is largely a consequence of increase in neuroinflammation and neurodegeneration.^37^ Cell death in TBI is associated with dysregulation of autophagy mechanisms, including mitophagy dysfunction.^38^, ^39^ In the present study, we found mild to moderate neuroprotective effects, increases in markers for autophagy, mitophagy neuroinflammation cell survival, pre‐ and post‐synaptic integrity, while decreasing markers of cell death following transplantation of MSCs‐IL‐10 and/or MSCs alone in a rat model of TBI. Furthermore, MSCs‐IL‐10‐transplanted rats displayed greater neuroprotective effects than rats which received MSCs alone.

Neuroinflammation is one of the key mechanisms associated with neuronal injury in TBI. Pro‐inflammatory cytokines, such as IL‐10, become down‐regulated in TBI, which trigger neuronal death.^17^, ^40^ Transplantation of MSCs has been shown beneficial therapeutic effects in different brain injury models,^41^ because they secrete many neurotropic factors, including cytokines, such as IL‐4, IL‐6, IL‐10, IL‐11 and IL‐13.^23^, ^42^, ^43^ Among them, IL‐10 is the most important because it exerts neuroprotective effects via suppressing the expression of various pro‐inflammatory cytokines, such as IFN‐γ, IL‐1β, IL‐2, IL‐6 and TNF‐α, as observed in stroke 23, 44 and in TBI.^42^, ^43^, ^45^, ^46^, ^47^ MSCs may secrete IL‐10 under specific conditions, such as inflammatory environments observed after brain injuries.^52^ Also, they may stimulate the cells surrounding the injury and trigger the secretion of IL‐10 and other neurotrophic factors.^52^ We genetically modified MSCs to secrete abundant IL‐10 and hypothesized that they may improve MSCs‐based cell therapy for TBI‐induced neuronal injury.^24^ We observed more neuroprotection exerted by transplants of MSC‐IL‐10 than by MSCs alone 24 (Figure S1).

We have used the rat‐controlled cortical impactor injury model and characterized the cell death in the cortex and in the hippocampal subfields, using multiple staining methods. A significant decrease in GFAP and Iba‐1 in the cortex after transplantation of both MSCs‐IL‐10 and MSCs alone in TBI rats (Figure S2) was observed, suggesting a reduction in neuroinflammation.^24^ Transplantation of MSCs alone was unable to decrease the number of GFAP‐IR cells in CA1 and CA3 areas of hippocampus. Similarly, transplantation of MSCs‐IL‐10, but not MSCs alone, significantly decreased the Iba‐I‐IR cells in the frontal cortex, (Figure S2),^24^ suggesting that MSCs‐IL‐10 exert greater anti‐inflammatory effects than MSCs alone, which was a finding supported by Nakajima and colleagues who used a mouse model of ischaemic stroke.^23^

We further investigated the autophagy mechanisms, which can provide cytoprotection,^48^, ^49^, ^50^, ^51^, ^52^, ^53^, ^54^ as seen in animal models of TBI and other neurological diseases.^39^ IL‐10 may induce autophagy or autophagy can enhance IL‐10 production, as reported previously.^55^ We have investigated several autophagy markers, such as Atg5, Atg7, Beclin‐1, LC3A/B, mTOR, p‐mTOR levels. We observed significant increases in Atg5 and Atg7 levels after transplantation of MSCs‐IL‐10 and MSCs‐GFP, indicating that MSCs‐IL‐10 or MSCs‐GFP can induce autophagosome formation (Figure 4), as reported by other investigators in animal models of AD,^31^ acute ischaemic stroke 23 and in TBI.^56^ Beclin‐1 levels, which are involved in autophagic cell death and apoptosis,^57^ were less in TBI rats (Figure 4), as also reported by Au and colleagues,^51^ but were restored by transplantation of MSCs‐IL‐10 and/or MSCs‐GFP indicating that the transplants exerted cytoprotective effects.^54^ Similarly, conversion of microtubule‐associated protein light chain‐3A/B‐I (LC‐3A/B‐I) to LC3A/B‐II, a reliable biomarker for autophagy,^58^ was significantly increased by MSCs‐IL‐10, but not by MSCs‐GFP (Figure 4), indicating that MSCs‐IL‐10 is a stronger inducer of autophagy. 52 Levels of p62, a marker for autophagy flux 57 and which directly binds to LC3, while its degradation causes decreased levels of LC‐II,^59^ were increased in TBI‐MSCs‐IL10 rats, but not by MSCs alone, suggesting that autophagy mechanisms were induced primarily by the MSCs‐IL‐10 transplant (Figure 4F). Increased p62 levels can indicate decreased autophagy, due to blocking the fusion of autophagy vacuoles with lysosome or by the inhibition of a later maturation step of autophagosome degradation.

Proteins which regulate autophagy mechanisms, such as phosphoinositol 3‐kinase (PI3K), Akt (protein kinase B) and mammalian target of rapamycin (mTOR) (PI3K/Akt/mTOR pathway),^59^ reported to be involved in the neuroprotection in cerebral injury,^56^ increase in the cortex and hippocampus of mice at 24 hours after TBI.^59^ Increased levels of p‐mTOR are the indicators of decreased autophagic responses.^60^, ^61^, ^62^ In the present study, we observed that the levels of PI3K (p85) and p‐Akt were decreased and p‐mTOR was up‐regulated by TBI, whereas transplantation of MSCs‐IL‐10, but not MSCs alone, increased their levels (Figure 4G‐I), suggesting autophagy was inhibited by TBI and transplantation of MSCs‐IL‐10 activated this pathway. Decreased levels of p‐mTOR correlated with increased levels of LC‐3A/B‐II (Figure 4E) and MSCs‐IL‐10 activated autophagy mechanisms by inhibiting PI3K/Akt/mTOR pathway but, further experiments are needed using mTOR inhibitor to confirm these findings.

In addition, mitochondria dysfunction, including reduction in mitochondrial respiration, increase in the production of ROS has been observed in TBI, triggers apoptotic cell death,^72^which can be mitigated by the induction of mitophagy. We found a significant increase in mitophagy markers, such as NIX, BNIP3, FUNDC1, PINK‐1 and HIF‐1α levels following transplantation of MSCs‐IL‐10 (Figure 6), but not by MSCs alone, suggesting that the damaged mitochondria were selectively degraded via mitophagy and that MSCs‐IL‐10 have a greater role in controlling mitophagy than by MSCs alone.

Molecular chaperones, or heat shock proteins (HSPs), are involved in cell death and survival by degrading small, misfolded proteins.^63^, ^64^ We observed that HSP90 was significantly up‐regulated (Figure S3) by TBI rats. Increased HSP90 has been reported to be involved in brain injury.^65^ Decreased levels of HSP90 by MSC‐IL‐10 was greater than by MSCs alone, suggesting that MSC‐Il‐10 has greater cytoprotective roles by down‐regulating its levels. In contrast, loss of HSP40 and HSP70 increases brain injury and death of neurons,^66^ whereas they can induce and arrest inflammation and improve neurological outcome.^67^ We found decreased levels of HSP40 and HSP70 in TBI rats and their levels were restored by MSCs‐IL‐10, not by MSCs alone (Figure S3), suggesting that MSCs‐IL‐10 may induce immunomodulatory and neuroprotective roles through HSPs. In addition, CMA markers, such as HSC70 and LAMP2A, were only modestly decreased in the TBI rats (Figure S3), but their levels were increased by both the transplanted groups, suggesting that CMA was activated to remove some of the debris generated by the transplanted cells.

There were less pyknotic or tangle‐like cells as revealed by CV stain (Figure 1), along with decreased neurodegeneration as shown by FJB stain (Figure 2) and reduced number of TUNEL‐positive cells (Figure 3) in MSCs‐IL‐10 transplanted TBI rats, relative to those in TBI + MSCs rats, suggesting the neuroprotective effects might be due to IL‐10. We also observed that there were decreased levels of Bax, caspase‐3, and cytochrome‐C after transplantation of MSCs‐IL‐10, but not by MSCs. Similarly, anti‐apoptotic markers Bcl2, synaptic markers PSD95 and synaptophysin were also restored by MSCs‐IL10, but not by MSCs alone. In addition, increased levels of p53 were also involved in TBI‐induced cell death,^68^ which was decreased by MSCs‐IL‐10, not by MSCs alone, suggesting that MSCs‐IL‐10 showed greater neuroprotective effects than MSCs alone, as reported previously.^24^, ^69^ Increased levels of these markers may be due to a decrease in neuroinflammation, due to an increase in anti‐inflammatory cytokines and neurotropic support, as well as increases in the autophagy mechanisms 70, 71. These findings also indicate that transplanted cells may secrete many other neurotropic factors,^23^ along with IL‐10, as reported by other investigators in mouse models of TBI 72, 73. Additional experiments are required to elucidate mechanisms of MSC‐IL‐10‐induced neuroprotection in TBI.

Overall, we found that the controlled cortical impact model of TBI in rats produced significant neurodegeneration and cell death in the cortex and in the hippocampus and that transplantation of MSCs‐IL‐10 provided greater neuroprotection than MSCs alone. Transplanted MSCs‐IL‐10 induced autophagy, mitophagy, molecular chaperones, regulated PI3K/Akt/mTOR pathway and influenced cell death and cell survival markers more efficiently than MSCs alone. Therefore, induction of autophagy mechanisms, using MSCs that overexpress IL‐10, may be an effective strategy for protecting the brain against TBI‐induced cell death.

## CONFLICT OF INTEREST

The authors declare that they have no competing interests to publish this research article.

## AUTHOR'S CONTRIBUTIONS

Study designed: PM and SP; PM and Data collection, analysis, interpretation and manuscript writing: PM. Histology and immunohistochemistry: NK. Animal surgery, tissue collection: SP, MA and AG. Manuscript editing and overall support: GD and JR. All authors approved the final manuscript.
