# Supplementary figures and images for: Transplantation of mesenchymal stem cells overexpressing interleukin‐10 induces autophagy response and promotes neuroprotection in a rat model of TBI
Source: J Cell Mol Med. 2019 Jun 4;23(8):5211–24. doi: 10.1111/jcmm.14396 (PMC6653779; doi:10.1111/jcmm.14396)

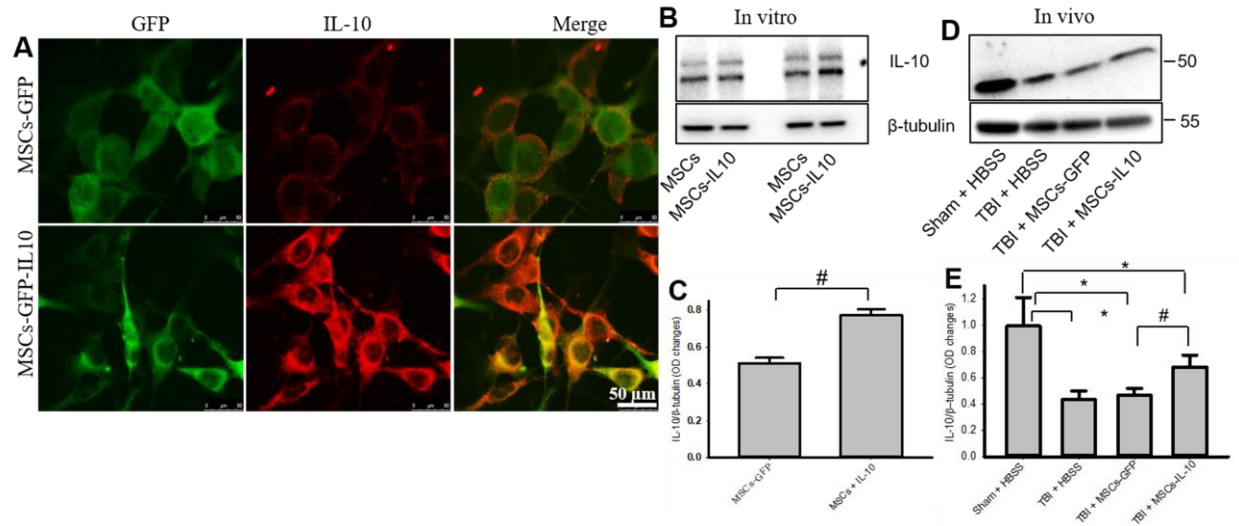

Supplement: Supplementary file 1 [file JCMM-23-5211-s001.pdf]

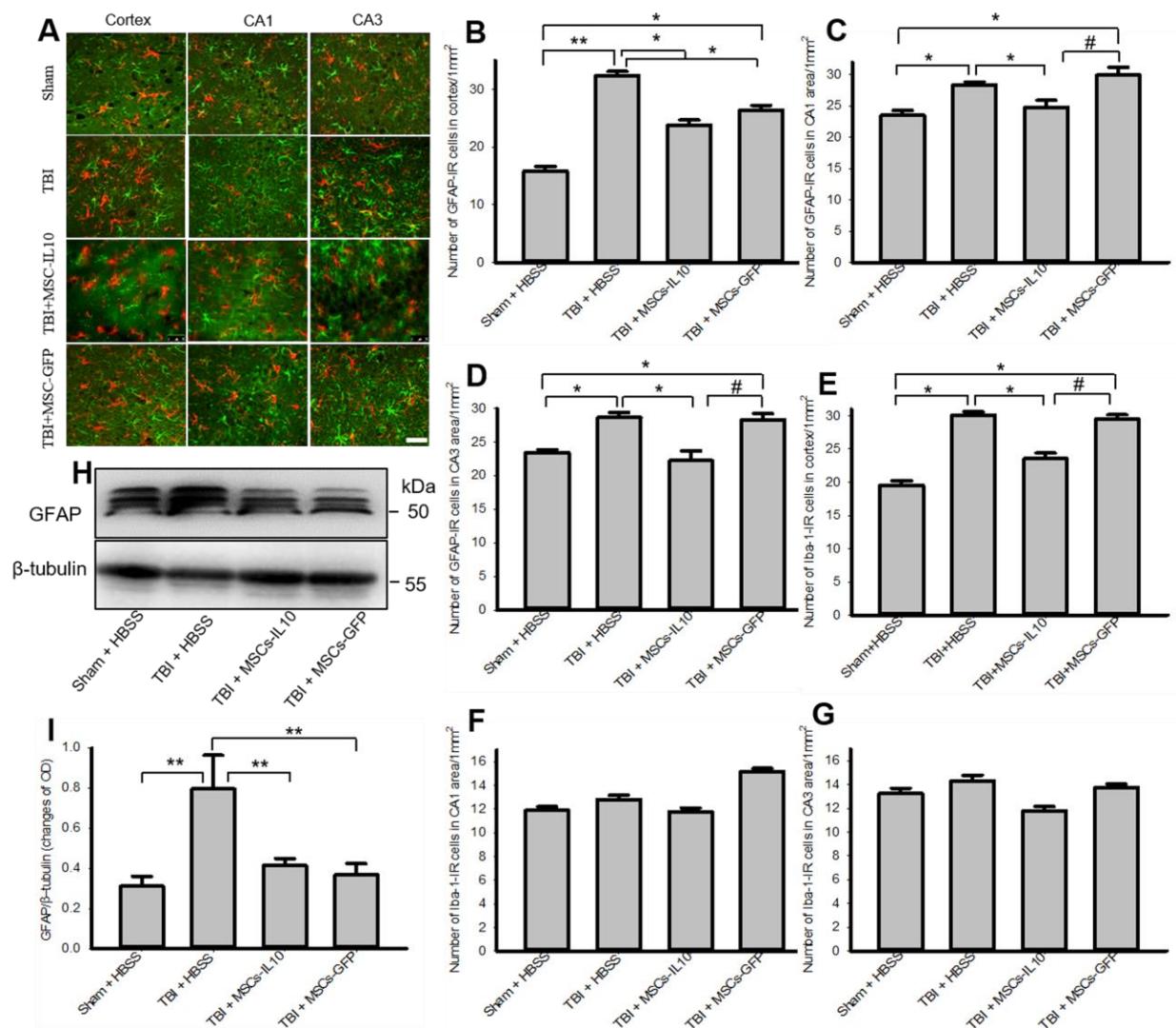

Supplement: Supplementary file 2 [file JCMM-23-5211-s002.pdf]

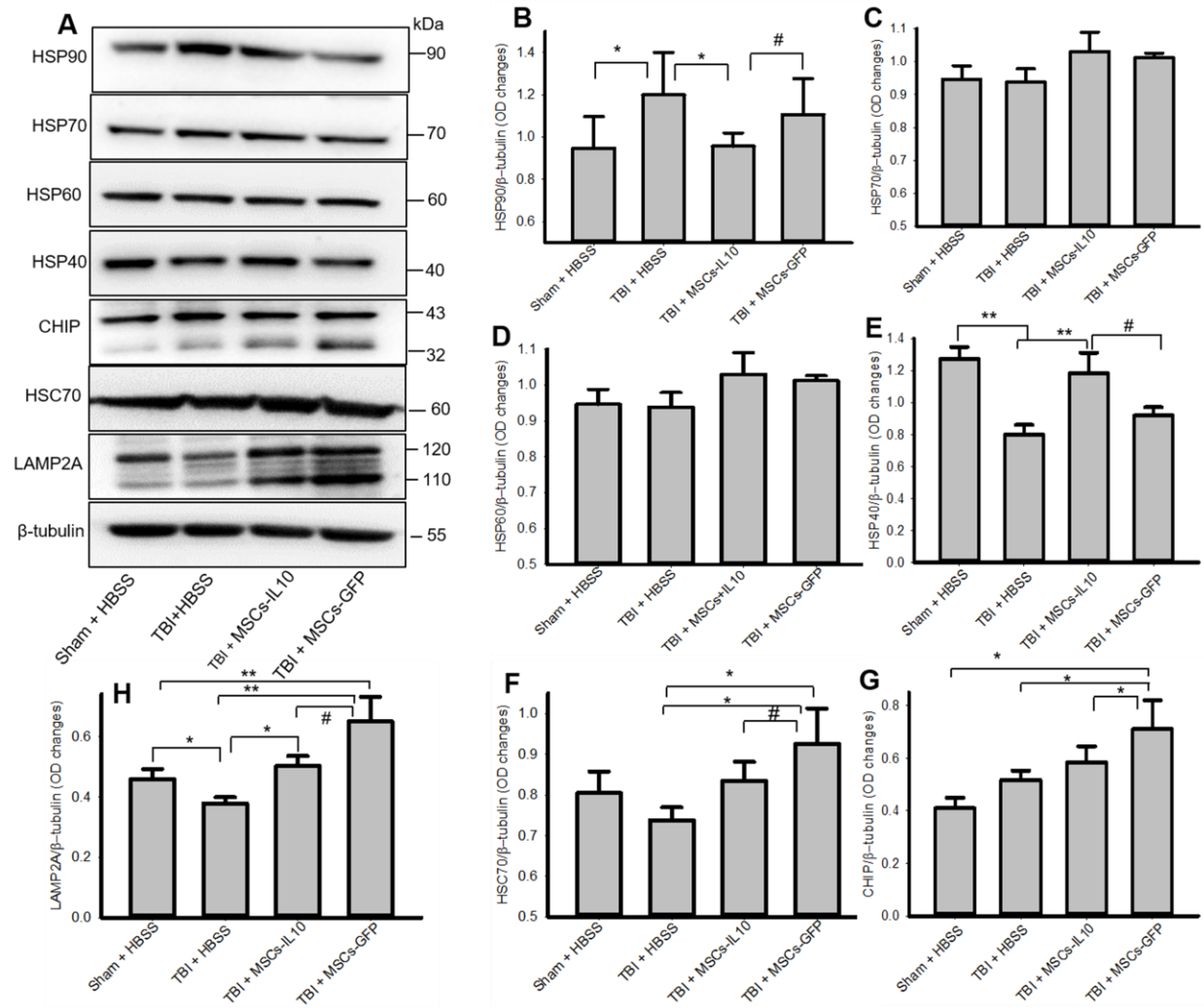

Supplement: Supplementary file 3 [file JCMM-23-5211-s003.pdf]
